# Adverse effects of gestational ω‐3 and ω‐6 polyunsaturated fatty acid imbalance on the programming of fetal brain development

**DOI:** 10.1111/jne.13320

**Published:** 2023-07-27

**Authors:** Valentina Cinquina, Erik Keimpema, Daniela D. Pollak, Tibor Harkany

**Affiliations:** ^1^ Department of Molecular Neurosciences Center for Brain Research, Medical University of Vienna Vienna Austria; ^2^ Department of Neurophysiology and Neuropharmacology Center for Physiology and Pharmacology, Medical University of Vienna Vienna Austria; ^3^ Deaprtment of Neuroscience Biomedicum 7D, Karolinska Institutet Stockholm Sweden

**Keywords:** arachidonic acid, dietary requirement, neurodevelopmental disorder, neuronal wiring, nutrient conversion

## Abstract

Obesity is a key medical challenge of our time. The increasing number of children born to overweight or obese women is alarming. During pregnancy, the circulation of the mother and her fetus interact to maintain the uninterrupted availability of essential nutrients for fetal organ development. In doing so, the mother's dietary preference determines the amount and composition of nutrients reaching the fetus. In particular, the availability of polyunsaturated fatty acids (PUFAs), chiefly their ω‐3 and ω‐6 subclasses, can change when pregnant women choose a specific diet. Here, we provide a succinct overview of PUFA biochemistry, including exchange routes between ω‐3 and ω‐6 PUFAs, the phenotypes, and probable neurodevelopmental disease associations of offspring born to mothers consuming specific PUFAs, and their mechanistic study in experimental models to typify signaling pathways, transcriptional, and epigenetic mechanisms by which PUFAs can imprint long‐lasting modifications to brain structure and function. We emphasize that the ratio, rather than the amount of individual ω‐3 or ω‐6 PUFAs, might underpin physiologically correct cellular differentiation programs, be these for neurons or glia, during pregnancy. Thereupon, the PUFA‐driven programming of the brain is contextualized for childhood obesity, metabolic, and endocrine illnesses.

## Graphical abstract

Cellular enrichment of the cerebral cortex during fetal brain development, including the timeline of neuronal and glial diversification, and the formation of neuronal networks. Major bioenergetic sources and potential modifications inflicted upon cellular identity were also marked.

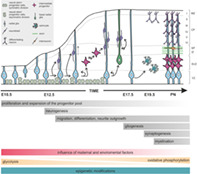

## 1 INTRODUCTION

Since David Barker first postulated a direct link between inadequate nutrition in early life and increased susceptibility to the onset of cardiometabolic pathologies in adulthood, the “fetal origins of disease” concept has evolved, and become broadly applied to a variety of conditions, including central nervous system (CNS) development.^1^ This is because the developing CNS is particularly vulnerable during intrauterine development to metabolic compromise given the exceptional energy demands of its many cell types that are being generated (including neurons, astroglia, microglia, oligodendroglia, vasculature), their protracted movements (migration), morphogenesis, and assembly into functional circuits. Thus, disruption of these processes by environmental factors will likely provoke long‐lived modifications to brain structure and, ultimately, function.^2^, ^3^ In principle, two models of developmental “programming” (that is, a lasting change to cell type identity and function) are distinguished: (i) direct “fetal programming”, when an intrauterine insult directly alters cellular identity to an extent that it manifests in somatic and/or mental illnesses later in life. Such diseases include schizophrenia, depression, and even the emergence of neurodegenerative diseases.^4^ (ii) An intrauterine insult evokes subthreshold changes only. Nevertheless, exposure to the same or a similar disruptor postnatally will provoke a disease phenotype. This scenario, often termed “dual‐hit hypothesis” can underpin the pathobiology of schizophrenia, psychosis, and panic disorder with maternal exposure to psychostimulants and/or illicit drugs recognized as lead candidates to evoke the “first hit” when taken during pregnancy. A second exposure to the same class of drugs (e.g., cannabis or synthetic cannabinoids) during adolescence can then be liable for neuropsychiatric diseases to manifest.^5^

Given the magnitude and pace of nutrient supply to and turnover within the developing CNS, the efficient exchange of nutrients with the maternal circulation is required to maintain the expansion and maturation of the neuronal connectome. Multiple gestational factors could affect CNS development including maternal psychosocial stress and infection,^6^ alcohol,^7^ illicit and/or prescription drugs,^8^ and maternal diet.^9^ Restrictive societal views, legislation, and even medications prevent fundamental changes to many of these maternal conditions during pregnancy. The foremost exception to these concerns is maternal food preference, that is, both the type of nutrition and its amount. Given the global obesity epidemic, which has by now spilled from the industrialized world to even developing countries, the study of how maternal nutrition affects fetal organ development has become a focus for public health regulators worldwide.^10^

The number of overweight and obese adults exceeded 1.9 billion by 2018, including ~42.5% of the US population alone.^11^ The percentage of women with pre‐pregnancy obesity also continues to rise. It is estimated that, globally, there are close to 39 million pregnancies per year complicated by maternal obesity and, in some countries, the estimated prevalence of overweight and obesity in pregnancy is over 60%.^12^ Notably, a simple mismatch between maternal supply versus fetal nutritional demand (that is, the gross level of energy and macronutrients) does not account for the harmful effects of maternal obesity on child development. Instead, obesity is closely linked to compositional changes in the diet, which are particularly pronounced when considering the amount and molecular identity of polyunsaturated fatty acids (PUFAs) in Western diets.^13^ Anecdotally, the oriental/Japanese diet is considered as the opposite of the Western diet and taken as healthy. It is not surprising, therefore, that children born to obese mothers have a high risk of being exposed to the undesired bioactive properties of fatty acids during prolonged periods of their intrauterine life.

Here, we provide a snapshot of contemporary concepts on the function of PUFAs during physiological brain development, and present examples on how they affect either neurons or (astro‐)glia, or both. We emphasize that the disbalance, rather than an absolute change, of any of the ω‐6 (n‐6)/ω‐3 (n‐3) PUFAs can adversely impact the developing CNS. Thus, we highlight a significant risk through imbalanced maternal nutrition given existing data to causally link the mother's nutritional status during pregnancy with the health status and long‐term postnatal prospect of their offspring (Table 1). The notion that disrupted fetal development might transcend to second and even third generations highlights the medical and socioeconomic importance of controlling obesity in pregnant women. Further mechanistic insights into the modulation of fetal brain development could advance the early diagnosis of negative outcomes, and the design of preventive and treatment measures.

**TABLE 1:** The impact of maternal diets imbalanced in ω‐6 PUFAs:ω‐3 PUFAs on child health.

Maternal factor	Child outcome	References
Obesity Gestational diabetes mellitus (GDM) Pre‐pregnancy body mass index (BMI)	↓ expressive language skills	Saros et al.^36^
	↓ memory performance	DeBoer et al.^37^
	↑ ADHD, ASD, schizophrenia symptomatology/risk, as well as of cognition impairment	Rivera et al.^39^
**ω**‐6/**ω**‐3 PUFA imbalanced diet	↑ risk of cognitive impairments	Bernard et al.^40^ Lopez‐Vicente et al.^41^
	↑ ADHD symptomatology/risk	Lopez‐Vicente et al.^41^ Galera et al.^44^

## 2 PUFAS: BIOCHEMISTRY IN A NUTSHELL

The human diet is the source of fatty acids, many of which are “essential fatty acids” that the human body is unable to produce. Once ingested, free fatty acids are absorbed from the gastrointestinal tract and transported by the bloodstream. At the cellular level, fatty acids do not only serve as energy sources but also as integral constituents of the cell membranes, thus affecting cell shape, size, protein localization, signal transduction, and survival. Additionally, fatty acid metabolites (e.g., endocannabinoids, endovanilloids, prostaglandins, and bile acids) shape both neuronal and glial responsiveness to peripheral hormones and peptides, as well as synapse formation, structural plasticity, and synaptic neurotransmission in the brain.^14^, ^15^, ^16^

**FIGURE 1:**
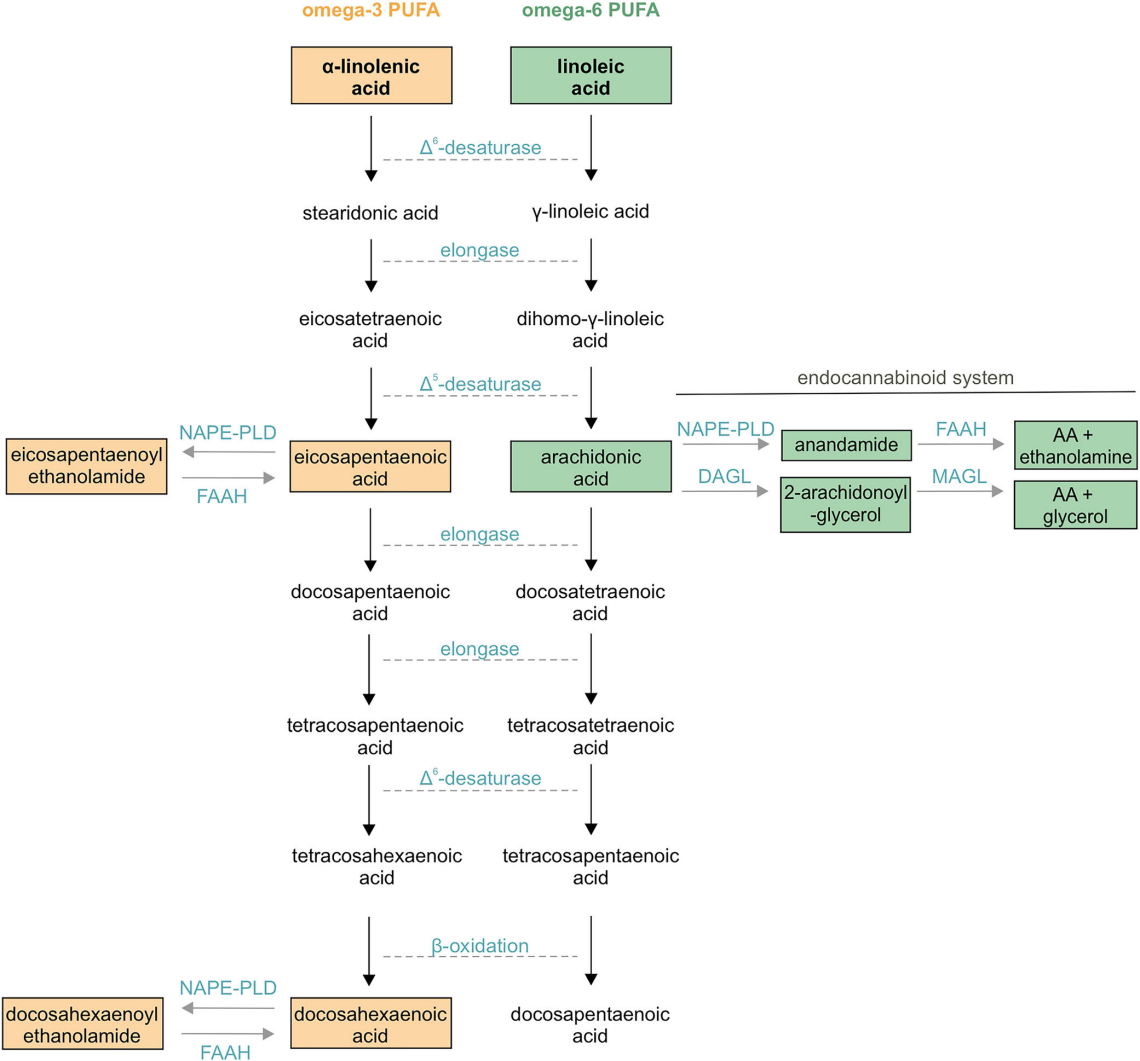
A summary of synthesis and metabolic pathways for both ω‐3 and ω‐6 PUFAs. Intermediates for both ω‐3 and ω‐6 PUFAs are produced through elongation and desaturation steps of their respective essential precursors, α‐linolenic acid (α‐LA) and linoleic acid (LA), respectively. Rate‐limiting enzymes were color‐coded in light blue.

Fatty acids are composed of a hydrocarbon chain with a methyl group and a terminal carboxyl group. A fatty acid is considered saturated if each carbon is joined to its neighbor by a single bond. If one or more double bonds exist, the fatty acid is considered unsaturated. Amongst unsaturated fatty acids, monounsaturated (one double bond) or polyunsaturated (more than one double bond; PUFA) entities are distinguished.^17^ Two main classes of PUFAs exist based on the position of their unsaturated carbon bonds at either their n‐3 or n‐6 position. The series of ω‐3/n‐3 and ω‐6/n‐6 PUFAs share much of their biosynthetic enzymes (Figure 1), hence conversion can occur. Linoleic acid (LA; 18:2 n‐6) and α‐linolenic acid (ALA; 18:3 n‐3) are the principal essential unsaturated fatty acids. LA and ALA differ in their physiological roles and can act in competition. LA can be converted into arachidonic acid (AA; 20:4 n‐6), which then serves as a precursor to n‐6 endocannabinoids/endovanilloids, prostaglandins, thromboxane, and leukotrienes, as well as their derivatives.^18^ ALA can be converted to two somewhat longer n‐3 PUFAs, eicosapentaenoic acid (EPA; 20:5 n‐3) and docosahexaenoic acid (DHA; 22:6 n‐3), which are both physiologically significant,^18^ and whose ethanolamine derivatives (DHEA, EPEA) are recognized as ω‐3 endocannabinoids.^19^

The mammalian brain contains large amounts of fatty acids, of which 50% are PUFAs.^20^ AA and DHA are particularly efficacious to alter the biophysical properties of cell membranes, transporters, and receptors, thus modulating neurotransmitter action ubiquitously in neural networks.^21^ During antenatal life, maternal nutrient intake, including that of dietary fatty acids, is the single‐most determinant of the formation of the brain's cytoarchitecture.^22^ Notably, the rate of PUFA conversion in the placenta and the fetus is limited.^23^ Therefore, mechanisms have evolved for preformed DHA and AA that circulate in the mother's blood to be transported across the placenta into fetal venous blood.^23^, ^24^ It is therefore not at all surprising that any change in alimentary PUFA intake correlates with neurochemical alterations (e.g., neurotransmitter, neuromodulator availability, surface abundance of receptors) in both the maternal and fetal brains,^25^ and exert lasting impact on cognitive performance in adult offspring.^26^ In the following sections, these observations are considered within the context of brain development, including physiological and disease states.

## 3 MATERNAL DIET AS A RISK FACTOR FOR MENTAL DISORDERS IN THEIR OFFSPRING

Both maternal obesity and undernourishment carry the risk of gestational complications,^27^, ^28^, ^29^ and affect fetal development as early as determining the number and differentiation stage of the cell mass in the blastocyst well before implantation.^30^ Here, we thematically review data from both human studies (Table 1) and rodent models (Table 2, 3, 4) on brain structure and behavioral outcomes in the offspring. We note that human obesity is modeled by feeding rodents with a high‐fat diet (HFD), generally resulting in 40%–60% of its energy equivalent from fat. When mimicking Western diets, fat content is enriched in ω‐6 PUFAs at the expense of some ω‐3 PUFAs. Conversely, oriental diets are mimicked by a disproportionate increase in ω‐3 PUFAs.^31^

**TABLE 2:** Outcomes of dietary manipulations with ω‐6 PUFA enrichment and/or ω‐3 PUFA deficiency; in vivo experiments.

Maternal factor	Brain region	Species	Molecular/cellular outcomes	Offspring behaviour	References
**ω**‐6^enr^/**ω**‐3^def^ diet	E14.5 cerebral cortex	Mouse	↓ thickness of neuronal layers, ↑ gliogenesis.	↑ anxiety‐related behaviour	Sakayori et al.,^88^
**ω**‐6 enriched diet	E18.5 cerebral cortex	Mouse	↓ cell migration, ↓ chromatin accessibility, ↑ DNA methylation.	↑ anxiety‐related behaviour	Cinquina et al.^9^
**ω**‐3 deficient diet	Adult hippocampus	Rat	↓ neuronal soma size.	not performed	Ahmad et al.,^159^
**ω**‐3 deficient diet	Adult hippocampus	Mouse	↓ dendritic spine density, ↓ dendrite length.	↓ spatial working memory	Madore et al.,^160^
**ω**‐3 deficient diet	E18.5 hippocampus	Mouse	↓ neurite outgrowth, ↓ synaptogenesis, ↓ synapses excitability.	not performed	Cao et al.,^137^
**ω**‐3 deficient diet	Adult hippocampus	Mouse	↑ *Bdnf* DNA methylation, ↑ apoptosis.	not performed	Fan et al.,^129^
**ω**‐3 deficient diet	Adult prefrontal cortex	Mouse	Changes in PUFA level in mouse brain, ↓ endocannabinoid function, ↓ synaptic plasticity.	↑ anxiogenic and depression‐like behaviour	Lafourcade et al.^90^
**ω**‐3 deficient diet	Not studied	Mouse	Not performed.	↑ anxiety‐related behaviour	Harauma and Moriguchi^100^
**ω**‐3 deficient and enriched diet	Adult prefrontal cortex	Rat	Disrupted endocannabinoid system.	↑ short‐term memory deficit, ↑ emotional reactivity following n‐3 enriched diet	Zamberletti et al.^93^
**ω**‐3 deficient diet	Adult prefrontal cortex, hippocampus, hypothalamus	Mouse	Altered endocannabinoid signalling, ↑ DHA levels in brain.	↑ anxiety‐ and depression‐like symptoms	Larrieu et al.^92^

**TABLE 3:** Outcomes of dietary manipulations with PUFA‐enriched diets; in vivo experiments.

Maternal factor	Brain region	Species	Molecular/cellular outcomes	Offspring behaviour	References
**ω**‐3 enriched diet	Not selected	ASD rat	not performed	↓ social and cognitive deficits.	Schiavi et al.^94^
**ω**‐3 enriched diet	Not selected	Mouse	↑ DHA levels in brain.	↑ memory performance.	Leikin‐Frenkel et al.^103^
**ω**‐3 enriched diet	Adult hippocampus	Rat	↓ microglia activation.	↓ bodyweight, ↓ depression‐like behaviour.	Peng et al.^95^
**ω**‐6 enriched diet	Adult hypothalamus	Mouse	↑ PKA activity in hypothalamus.	↑ aggressiveness, ↑ exploratory activity.	Raygada et al.^85^
**ω**‐6^enr^/n‐3^def^ diet	Adult midbrain	Mouse	↑ mesolimbic dopamine release.	↑ food intake.	Sakayori et al.,^86^
**ω**‐6 enriched diet	Not selected	Mouse	Not performed	↑ anxiety‐related behaviour, ↑ autistic‐like sociability deficit,	Jones et al.^87^
**ω**‐6 enriched diet	Not selected	Mouse	Not performed	↑ depression‐like behaviour in male but not in female mice.	Yau et al.^117^
**ω**‐3 deficient and enriched diet	Not selected	Rat	Not performed	↓ maternal care behavior, ↓ USVs (following both diets).	Colucci et al.^99^
**ω**‐3 enriched diet	Not selected	ASD mouse	Not performed	↓ communication deficits of ASD mutant female mice (but not in males).	Nolan et al.^121^
High‐fat diet	Adult hippocampus, amygdala	rat	↑ corticosterone receptor expression, altered gene expression for pro‐ and anti‐inflammatory cytokines.	↑ anxiety behavior.	Sasaki et al.^116^

Abbreviations: ASD, autism spectrum disorders; PUFA, polyunsaturated fatty acid.

**TABLE 4:** Outcomes of dietary manipulations with high fat diets and specific PUFA supplements; in vivo experiments.

Maternal factor	Brain region	Species	Molecular/cellular outcomes	Offspring behavior	References
High‐fat diet perinatal	Not studied	Rat	Not performed	Lacking the typical decrease in USV numbers, shorter calls, ↑ one‐frequency‐sweep calls in females, ↑ two‐frequency‐sweep calls in males.	Abuaish et al.^111^
High‐ and low‐fat diets, perinatal	Not studied	Mouse	↑ macrophage toll‐like receptor 4 signaling in male fetal and adult brain, ↓ serotonin levels in male brain.	↓ sociability in females, ↓ non‐social reward in males, ↑ escape in males.	Ceasrine et al.^112^
High‐fat diet, perinatal	Hypothalamus	Rat	Altered genes expression for ECM proteins.	Not performed	Barrand et al.^115^
High‐fat diet, perinatal	Amygdala, hippocampus	Rat	↑ corticosterone receptors in amygdala, altered pro‐ and anti‐inflammatory cytokines, ↓ corticosterone levels.	↑ anxiety behavior.	Sasaki et al.^116^
LA‐enriched and LA‐decreased diet	Not studied	Rat	↓ ω‐3 PUFA and ALA after LA‐enriched diet, ↑ ω‐6 PUFA, LA and AA after LA‐enriched diet, ↓ adiponectin in females, ↑ circulating leptin in females.	Not performed	Shrestha et al.^118^
DHA supplement	Not studied	Mouse	↑ DHA, EPA, DHEA levels in both brain and plasma.	Not performed	Wood et al.^101^
**ω**‐3 deficient and enriched diet	Cerebral cortex, hippocampus	Rat	↓ cortical CB1R and ↑ cortical and hippocampal CB2R in mother's brain after ω‐3 deficient diet, ↓ hippocampal CB1R and CB2R in neonates after ω‐3 deficient diet, ↑ hippocampal CB2R in neonates after ω‐3 deficient diet, ↑ cortical level of both DHEA and EPEA and ↓ cortical level of 2‐AG after ω‐3 enriched diet, ↑ PKA and ERK phosphorylation.	Not performed	Isaac et al.^102^
**ω**‐3 enriched diet	Not studied	Mouse	Not performed	↓ number of calls in females, rescue of ASD‐like deficits in females.	Nolan et al.^121^ Pietropaolo et al.^122^

Abbreviations: 2‐AG, 2‐arachidonoyl glycerol; AA, arachidonic acid; DHA, docosahexaenoic acid; CB1R/CB2R, type 1/2 cannabinoid receptor; DHEA, dehydroepiandrosterone; ECM, extracellular matrix; EPA, eicosapentaenoic acid; PKA, protein kinase A; PUFA, polyunsaturated fatty acid.

### 3.1 Human data on maternal diet affecting the offspring's intellectual ability

Weight gain is an essential part of a healthy pregnancy to support fetal growth, placental development, and the production of amniotic fluid. Therefore, it is only chronic overweight and obesity, defined by the World Health Organization as a body mass index (BMI) of 25–29.9 kg/m^2^ and≥30 kg/ m^2^, respectively, that are seen as health risks. High BMI in pregnant women is associated with gestational diabetes (hyperglycemia, insulin resistance), and pre‐eclampsia, pathologies that increase both maternal and infant mortality and morbidity.^32^, ^33^ During pregnancy, the fetus(‐es) of obese mothers are particularly prone to overgrow, have altered body composition, and display neural tubes defects.^27^, ^34^ Long‐term consequences for children born to obese mothers include cardiovascular disease, metabolic syndrome, diabetes, cancer, chronic inflammatory disorders, and psychiatric diseases.^35^ Longitudinal follow‐up of 1‐ and 2‐year‐old children born to overweight or obese mothers substantiated a link between gestational diabetes versus reduced memory performance and delayed language skills, maternal obesity versus cognitive, language, and motor skills^36^, ^37^ (Table 1). Developmental deficits of the CNS persisted in school age children and were classified as mild cognitive impairment.^38^ However, food addiction, anxiety, depression, attention deficit hyperactivity disorder (ADHD), autism spectrum disorders (ASD), and schizophrenia are amongst the mental health disorders that children of obese (and/or diabetic) mothers most frequently display.^39^

Whilst maternal BMI is a predictor of the outcome of pregnancy and even lactation; recent studies highlighted that a shifted maternal dietary ω‐6/ω‐3 PUFA ratio is closely associated with reduced cognitive performance in the offspring.^40^, ^41^ Based on evolutionary principles, an “ideal ratio” of ω‐6/ω‐3 PUFAs ranges between 1:1 and 2:1 in the brain. This is also suggested to be the correct target ratio for daily intake.^31^, ^42^ When this ratio deviates in either direction, adverse effects might ensue. For example, the Norwegian Mother and Child Cohort Study demonstrated that the intake of a diet classified as “unhealthy” during pregnancy because of its high content of processed meat, refined cereals, salty snacks, and sweet drinks increased the prevalence of externalizing problems among infants at 12–18 months of age.^43^ Likewise, results from a French mother–child cohort revealed that maternal preference for a Western diet positively correlated to the offsprings' ADHD symptoms at ages ranging from 2 to 8 years.^44^ When the prenatal ratio of ω‐6:ω‐3 PUFAs was found shifted towards ω‐6 PUFAs in cord plasma, subclinical symptoms of ADHD appeared at 7 years of age.^41^ Strikingly, symptom scores for ADHD increase by ~13% per unit change in the ω‐6: ω‐3 PUFA ratio; an association that is likely driven by low DHA levels.^41^ Conversely, maternal intake of a diet enriched in fruit, whole‐grains, vegetable, and fish was associated with improved cognitive development (e.g., visual spatial skills), intelligence, and executive function at both early and mid‐childhood.^45^

Yet maternal effects of the diet are not restricted to intrauterine life. Alike the cellular reprogramming effects of psychoactive drugs, for example, Δ^9^‐tetrahydrocannabinol (THC),^46^ delivered by breast milk to infants, a shifted (1:8.4) ratio of ω‐6 and ω‐3 PUFAs negatively influenced the outcome of the parent‐reported Communicative Development Inventory and Ages and Stages Questionnaire for 2‐year‐old infants against never‐breastfed children.^47^, ^48^

These data suggest that maternal dietary preferences significantly affect both pre‐ and postnatal offspring development. Here, we first discuss maternal determinants of PUFA‐driven fetal outcomes, such as obesity‐induced comorbidities, changes in placental transfer, and the altered composition of breast milk before providing mechanistic insights in cellular programming events for neurons and astroglia.

#### 3.1.1 Obesity‐related obstetric complications

In obese individuals, increased food intake alters the circulating levels of nutrients, with the resultant increase in the amount of adipose tissue affecting the plasma levels of hormones controlling energy homeostasis, growth factors, and inflammatory mediators.

In overweight and obese pregnant women, plasma levels of adiponectin are lower than in normal weight women (reported as 8.4 ± 5.3 vs. 12.6 ± 6.0 ng/mL), thus increasing fat mass, insulin resistance, glucose production, and birth weight.^49^, ^50^ In contrast, the plasma concentration of leptin, a peptide hormone encoded by the obese (*ob*) gene, is significantly increased in both overweight and obese mothers, as compared to normal weight women (33.4 ± 14.8 vs. 23.0 ± 10.8 ng/mL). This increase is suggested to predict the development of pre‐eclampsia.^49^

During pregnancy, obese women are prone to develop hyperinsulinemia, glucose tolerance, and an altered lipid profile.^51^ Even though the levels of circulating lipids are per se increased in healthy pregnancies in normal weight women, obesity lowers the levels of high‐density lipoprotein (HDL), particularly in the first trimester (13.0 ± 0.9 mg/mL; rage: 3.8–30.8). Conversely, obese pregnant women display elevated triglyceride (TG) levels in both the second and third trimesters, which is a known risk factor for post‐partum hypertriglyceridemia.^52^ TGs are hydrolyzed to non‐esterified (free) fatty acids, whose levels remain elevated in the plasma of obese pregnant women.^51^

Maternal obesity also exacerbates the mild pro‐inflammatory state known to be associated with any normal pregnancy.^53^ In doing so, it increases the plasma levels of, for example, interleukin‐6 and tumor necrosis factor α, proinflammatory cytokines that stimulate placental trophoblast fatty acid accumulation leading to excess lipid and amino acid transport through the placenta and energy metabolism within.^54^, ^55^, ^56^ Cumulatively, these observations suggest that obesity adversely affects pregnancy outcomes.

#### 3.1.2 Placental function in obese mothers

The placenta acts as an essential interface for the transport of nutrients and hormones necessary for fetal growth. Given the poor ability of the fetus to synthesize non‐essential PUFAs, the levels of AA and DHA in the fetus strictly depend on active transport across the placenta.^57^ To do so, the functional unit of the placenta is the trophoblast villous tree, which incorporates fetal blood vessels, and covered by syncytiotrophoblasts, which provide the primary plasmalemmal interface of the placenta with transporters used for the maternal‐fetal active exchange of glucose, amino acids, and lipids.

During gestation, TGs and fatty acids are transported through specific placental transport systems, and binding proteins. In normal weight pregnancies, fetal fatty acid levels are significantly lower than in maternal plasma (fetal/maternal ratio: ~1:3).^58^ In particular, the PUFA profile is different between the maternal and fetal circulation, with AA and DHA increased versus LA and ALA decreased in fetal plasma as compared to the maternal counterpart.^58^

Maternal obesity alters the supply of fatty acids through the placenta by affecting its transport mechanisms.^59^ Notably, Segura et al.^60^ reported elevated PUFA content in the placenta in obese pregnant women versus normal weight women. In particular, EPA was found increased in the phospholipid fraction of placental tissues of obese women. This increase is likely a result of the higher expression of both FATP6, a fatty acid transport protein, and FAT/CD36, a fatty acid translocase. ALA, LA, AA, and DHA remained unchanged, suggesting the selective mobilization and utilization of specific PUFAs.

It is generally acknowledged that higher fetal adipogenesis shall be the result of an increased cross‐placental lipid transfer in obesity.^61^ However, when maternal obesity reaches extreme severity, the ability of the placenta to transport any nutrient might become severely compromised or fail, causing the cessation of delivering PUFAs to the fetus.^62^ Thus, one might suggest a correlation between maternal obesity and placental function, which can though progress from increased transfer to an acute impairment of nutrient supply. Identification of the molecular trigger(s) of this phenomenon will benefit from further research.

#### 3.1.3 Maternal diet and breastfeeding

The composition and availability of breast milk are the single‐most determinants of neonatal development.^63^ Breastfeeding is viewed as healthy for both the baby and the mother, with its health benefits likely enduring into childhood and manifesting as improved cognitive development.^64^ As such, medical guidelines recommend breastmilk to be the only source of nutrients for the first 6 months of life; and continued up to 2 years when possible.^63^ This is because human breastmilk covers most of the energy (ketogenic source) and fatty acid requirements of the developing infant(s). The fat content of human breastmilk is ~3.8–3.9 g/100 mL,^65^ yet with a composition changing over time due to the type of milk produced (that is colostrum [produced for ~5 days after giving birth], transitional milk [TM], mature milk [MM]), the feeding stages (foremilk, hindmilk), and the dietary habits of the mothers during pregnancy and lactation.^66^

The overall PUFA content of breastmilk changes throughout lactation: it is highest in the colostrum (21.47 g/100 mL) and gradually decreases as the milk matures (TM: 18.82 g/100 mL, MM: 18.53 g/100 mL). Likewise, the mean ω‐6 PUFA content becomes gradually reduced (colostrum: 19.88 g/100 mL, TM: 17.56 g/100 mL, MM: 17.28 g/100 mL).^66^ Among the ω‐6 PUFAs, LA is the most prevalent, amassing ~10% of the total fatty acid content of breast milk. Approximately 30% of LA present in the milk is directly transferred from the diet, while the rest (70%) is generated by adipose tissue. Amongst ω‐3 PUFAs, ALA dominates yet at a concentration ~10‐fold lower than LA,^65^ thus resulting in a shifted ω‐6: ω‐3 PUFA ratio. Notably, ~65% of ALA is of dietary origin with only ~35% produced by the body itself.

Particular emphasis shall be directed towards AA and DHA because their derivatives (endocannabinoids, DHEA/”synaptamide”^67^) are critical for axonal growth and synaptogenesis.^68^, ^69^, ^70^ Both AA and DHA have a propensity to accumulate in the tissues of both fetuses and infants during the third trimester of pregnancy and in the first months of life, respectively.^23^, ^24^ The latter observation is despite that their concentration is gradually reduced in human milk as lactation progresses.^66^ One way to increase the conversion of ALA to DHA is to supplement the mothers' diet. This, however, only benefits plasma DHA levels during pregnancy, and thus its placental transfer, but less so the DHA concentration in the mother's milk.^71^, ^72^, ^73^ This phenomenon is likely due to the low efficiency of the human body to convert ALA into bioactive derivatives, such as DHA and EPA. Accordingly, clinical studies in Greece, China, and US reported a positive correlation between dietary DHA quantities during lactation and the amount of DHA excreted into the breast milk,^74^, ^75^, ^76^, ^77^ and led to a daily recommended dose of 250–500 mg/day for both DHA and EPA during pregnancy.^78^, ^79^ These guidelines are on the backdrop of high geographical variations in DHA intake, ranging from 30 to 180 mg/day in women living in inland and coastal areas, respectively.^80^ In sum, the above data show that dietary preferences can influence the composition of breast milk.

The impact of maternal weight on the fat content of breastmilk is an important aspect of early postnatal tissue programming. Maternal obesity skews the ω‐6: ω‐3 PUFA ratio as early as in the colostrum, with a discrepancy that persists until the MM.^81^ In addition, a close correlation exists between delayed lactogenesis and the maternal BMI categories: from 31% among women in the normal BMI range, to 43% among women in the overweight BMI range and 52% among women in the obese BMI range.^82^ This acutely increases the risk of excess neonatal weight loss. Obese women are also more likely to have shortened periods of breastfeeding.^83^ Thus, we conclude that maternal obesity poses a significant risk for the success of adequate nutrition for newborns when considering breastfeeding as the primary route of nutrition.

### 3.2 Modified experimental diets during pregnancy and their developmental outcomes

Animal studies are critical in interrogating the mechanisms by which maternal diets program the brain, and consequently trigger behavioral dysfunction in affected offspring. Animal models using imbalanced ω‐6: ω‐3 PUFA diets are well documented (Table 2, 3, 4) to produce long‐lasting changes in the brain functions of affected rodent offspring.^84^ As early as 1998, the effect of a HFD enriched in ω‐6 PUFAs (43% energy derived from fat, high in ω‐6 vs. 16% in control diet) on offspring behavior was documented, including altered escape behaviour, aggressiveness, and locomotor activity.^85^ Ever since, a series of studies showed (Table 2, 3, 4) that the consumption of experimental diets high in ω‐6 but low in ω‐3 PUFAs by pregnant mice (i) increases the consumption of palatable foods by their offspring by impinging on mesolimbic dopamine neurotransmission,^86^ (ii) provokes anxiety and depression‐like behavior traits^9^ and (iii) autistic‐like sociability deficits^87^ in adult offspring. In developmental models, maternal consumption of diets with a high LA (n‐6):ALA (n‐3) ratio translated into a significant increase in ω‐6: ω‐3 PUFA ratio in the offspring's brain, and contributed to slowed CNS development.^88^, ^89^, ^90^, ^91^ Indeed, maternal diets particularly high in LA and poor in ALA were detrimental for long‐term synaptic plasticity, particularly synaptic depression mediated by AA‐derived endocannabinoids, in, among other areas, the prefrontal cortex, nucleus accumbens,^90^, ^92^ and hypothalamus^92^ of adult offspring. These changes behaviorally manifested as impaired emotional behavior, cognition, and memory.^93^

Experimental models also reinforced the health benefits of ω‐3 PUFA supplements (e.g., diet of 6% fat enriched in ALA) during gestation: ω‐3 PUFAs rescued social and cognitive deficits of ASD‐like traits in rats.^94^ More specifically, EPA (and also DHA though more moderately) was efficacious in attenuating chronic unpredictable mild stress‐induced weight loss and depression‐like behaviors, and increased exploratory drive in novel environments.^95^ Thus, the physiologically adequate brain and cognitive development of the offspring are programmed in utero by maternal nutrients.

Alike for humans, both the quantity and quality of maternal care during early life stages determines emotional behavior in adult animals.^96^, ^97^ Pups look for and respond to their parents by ultrasonic vocalizations (USV).^98^ Experimental diets enriched in either LA (ω‐3–deficient diet) or ALA (ω‐3–enriched diet) decreased maternal care behaviors (e.g., nest building, arched, blanket, and passive nursing, licking, pup retrieval, breast grooming) whilst promoting non‐maternal behaviors (feeding, exploring, self‐grooming), and changed the pattern and subtypes of USVs of pups deprived of their mothers. Thus, long‐lasting behavioral disturbances ensued in the experimental offspring.^99^

#### 3.2.1 Imbalanced ω‐6: ω‐3 PUFA diets impact brain lipid composition

Experimental models allow unprecedented analytical accuracy in determining PUFA composition of the developing brain at both prenatal and postnatal time‐points. To manipulate the ω‐3 PUFA content of the brain, an experimental diet deficient in ω‐3 PUFAs (LA: 74.4% of total lipids; ALA: 0.3% of total lipids; ω‐6/ω‐3 ratio 121.4 ± 1.9) decreased its DHA content,^90^, ^92^ and allowed associations between low DHA levels and anxiety‐ and depressive‐like symptoms,^90^, ^92^ as well as impaired learning.^91^, ^100^ It is noteworthy that a lowered maternal intake of DHA directly led to a reduction in DHEA (“synaptamide”) in the fetal brain,^101^ which could exceed an 80% loss when ω‐3 PUFAs were removed from the maternal diet.^69^, ^102^ In contrast, enrichment of the maternal diet in ω‐3 PUFAs increased DHA, DHEA, and EPEA levels in the cerebrum of neonatal offspring,^102^ and improved their memory performance later in life.^103^ These data corroborate observations in humans on a direct link between maternal and fetal circulation and placental fatty acid transfer, and the ability of PUFAs to modify adult behaviors with ω‐6 PUFAs and ω‐3 PUFAs being antagonistic (risk vs. resilience) factors, respectively.

### 3.3 Sex‐specific sensitivity and outcome severity in fetal brains

Environmental factors (be these illicit drugs,^104^, ^105^ stress,^106^, ^107^ maternal immune activation,^108^, ^109^ or prenatal malnutrition^110^) can have sex‐specific outcomes, particularly when priming for neurodevelopmental vulnerabilities. Do dietary PUFAs also act in a sex‐specific manner?

As discussed above, HFD during pregnancy can increase tissue levels of pro‐inflammatory cytokines in utero. This is a fundamental feature of developmental pathobiology, mediated by the pattern recognition receptor toll‐like receptor (*Tlr*)‐4 in the placenta and fetal brain, equally affecting male and female offspring. Changes in USVs emitted by infant offspring were also changed in both sexes, and compromised maternal pup retrieval and care behaviors in HFD dams.^111^ Yet, the specific behavioral phenotypes that adult offspring exhibit are sex‐specific: females have significantly decreased sociability scores, whereas males had diminished non‐social reward behaviors, and increased escape responses.^112^ Equally sex‐determined is the increased vulnerability of male offspring because of the altered epigenetic control of gene transcription in the developing brain,^113^ which could limit spatial learning and memory.^114^ In turn, female offspring show more pronounced stress pathobiology in adulthood, with altered gene expression seen in both the hypothalamus and amygdala.^115^, ^116^

An added level of differential responses are seen when using specified diets: an ω‐6 PUFA‐enriched diet (>6% of energy from LA vs. ~1.4% in control) offered during pregnancy, lactation, and/or the post‐weaning period increased depression‐like behavior in male but not female offspring,^117^ as measured in the forced swim and open field tests. These data also support the notion that the life‐long intake of LA can pose a significant risk for depression onset, with a greater susceptibility in males. Although mechanistic understanding of these phenomena is as yet fragmented, sex‐specific differences in the expression of genes for lipid metabolism might underpin the adverse effects of LA administered in utero and neonatally.^118^ However, in a mouse model of fragile X syndrome and ASD, the loss of the *Fmr1* gene recapitulated behavioral alterations in humans, including hyperactivity, anxiety, cognitive deficits, and and changes in sensorimotor gating.^119^, ^120^ When exposing *Fmr1* knock‐out mouse dams to an ω‐3 PUFA‐rich diet, the rescue of ASD‐like deficits in female but not male offspring was recorded, including improved emotionality, social interaction, and non‐spatial memory.^121^, ^122^ Cumulatively, these data suggest that ω‐6 and ω‐3 PUFAs can differentially impact the behaviors of female and male offspring born to dams on experimental diets.

### 3.4 Epigenetic mechanisms underpin PUFA action

Epigenetic regulation is a versatile and powerful mechanism to control cellular differentiation programs, and to maintain altered cell‐states at the long term. A robust line of research highlights epigenetic mechanisms, e.g., DNA methylation, histone modifications, and non‐coding RNAs, as central to brain development.^123^, ^124^ Indeed, the maternal diet can re‐model epigenetic patterns during prenatal development, with the incorporation of repressive marks being likely negative for long‐term outcomes in the offspring.^125^ For ω‐3 PUFAs, the activation of transcription factor cascades is likely^126^, ^127^ as a result of the modulatory action of epigenetic regulators, such as DNA (de‐)methylation globally or through gene‐specific promoter modifications.^128^, ^129^ As such, reduced DNA methyltransferase (DNMT) 3A and 3B activity was proposed as a potential molecular underpinning of variations in placental DNA methylation upon insufficient ω‐3 PUFA availability. Particularly, ω‐3 PUFAs could sustain or even increase *Bdnf* transcription (encoding brain‐derived neurotrophic factor), a hypothesis derived from loss‐of‐function studies with ω‐3 PUFA‐deficient diets increasing the methylation of the *Bdnf* promoter.^129^ Since *Bdnf* signaling is central to neurogenesis and neuronal differentiation (including neuronal survival and synaptogenesis), diet‐induced modifications to the availability of this neurotrophin can indeed alter brain architecture. Alternatively, ALA and DHA affect neurogenesis by modulating miRNA expression.^130^ Finally, ω‐6 PUFA‐derived endocannabinoids can disrupt neuronal differentiation by desensitizing CB_1_ cannabinoid receptors (CB_1_Rs), with a subsequent hypermethylation and reduced chromatin accessibility of target genes.^9^ Overall, epigenetic mechanisms seem fundamental for the sex‐specific and long‐lasting ability of dietary PUFAs to reprogram cellular identity and differentiation trajectories.

## 4 MECHANISTIC INSIGHTS IN PUFA ACTION ON NEURONAL DEVELOPMENT

**FIGURE 2:**
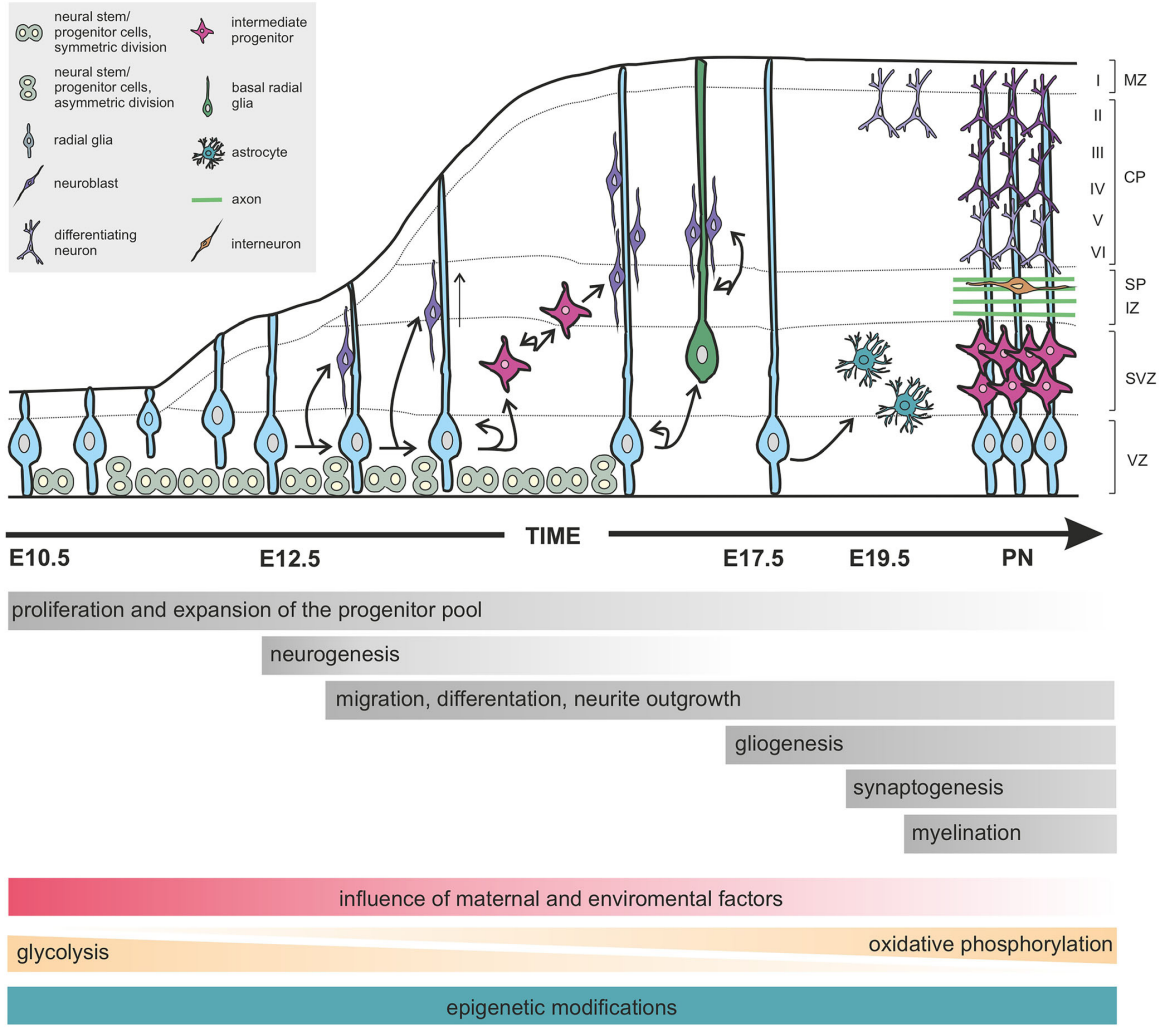
Neural progenitor subtypes and neurogenic stages during development of the cerebral cortex in mice. Cortical neurogenesis commences with the intense proliferation of progenitor cells in the ventricular zone (VZ) by embryonic day 10.5. During an initial phase, stem cells expand symmetrically, thus allowing the clonal expansion of radial glia. These progenitors then divide asymmetrically to generate neurons and glia, either directly or indirectly through intermediate progenitor cells or basal radial glia (alike tanycytes in the hypothalamus). Post‐mitotic neurons then migrate towards the pial surface and complete their differentiation in the cortical plate (CP). Neurogenesis is followed by a gliogenic phase with the generation of, for example, astrocytes. Arrows indicate lineage relationships as demonstrated by time‐lapse imaging and/or retroviral lineage tracing. CP, cortical plate; IZ, intermediate zone; MZ, marginal zone; SP, subplate; SVZ, subventricular zone; VZ, ventricular zone.

While the exact mechanisms by which ω‐3 and/or ω‐6 PUFAs affect specific stages of brain development are often incompletely understood, consensus exists on the many neurodevelopmental processes regulated by PUFAs, including, but not limited to, neural progenitor proliferation, neuroblast migration, differentiation (including both neuritogenesis and synaptogenesis), axonal myelination, and synapse pruning by microglia^84^, ^131^ (Figure 2 and Table 5). Moreover, a rapidly evolving field focuses on the liberation of PUFAs by exosomes as a means of itnercellular communication, for which we refer to a recent review.^132^ Notwithstanding, ω‐3 and ω‐6 PUFAs were contrasted as “good” versus “bad” for decades, a description we view as misleading because it does not accurately reflect their dietary ratios but emphasizes their effect sizes instead.

**TABLE 5:** Cellular processes affected by ω‐6 and ω‐3 PUFAs during neurogenesis and neuronal differentiation; in vitro experiments.

Experimental procedures	Species	Cellular outcomes/phenotypes	References
DHA, EPA and AA treatment of neural stem cells	Mouse	EPA and DHA increase endocannabinoid levels, EPA increases stem cell proliferation via endocannabinoid signalling, ↑ cell fate decision, ↑ cell survival.	Dyall et al.^127^
DHA, EPA and AA treatment of neural stem cells	Rat	DHA and EPA (but not AA) increase neuronal differentiation, DHA and EPA regulate the expression of basic helix–loop–helix transcription factors, DHA controls cell fate.	Katakura et al.^126^ Katakura et al.^136^
DHA and AA treatment of neural stem cells	Rat	AA and DHA promote the maintenance of neurogenic potential, DHA promotes the maintenance of gliogenic differentiation.	Sakayori et al.^135^
DHA, AEA, and DHEA treatment of primary cortical neurons and neural stem cells	Mouse	DHEA promotes neurite outgrowth and synaptogenesis, DHEA acts through the GRP110 receptor, DHEA induces cAMP production.	Lee et al.^141^
Pyramidal cell cultures and in vivo (transgenic mice)	Rat and mouse	AA‐derived endocannabinoids regulate neural progenitor proliferation and lineage commitment.	Galve‐Roperh et al.^148^ Molina‐Holgado et al.^150^ Mulder et al.^147^ Keimpema et al.^70^
Cultured interneurons and pyramidal cells and in vivo (transgenic mice)	Rat and mouse	AA‐derived endocannabinoids regulate the migration and differentiation of immature neurons.	Berghuis et al.^146^ Berghuis et al.^144^ Mulder et al.^147^
Cultured interneurons	Rat and mouse	Endocannabinoids regulate synaptic communication.	Berghuis et al.^146^ Berghuis et al.^136^
DHA treatment of primary hippocampal neurons	Mouse	↑ neurite outgrowth and improved synaptogenesis, ↑ synaptic protein expression.	Kim et al.^138^

Abbreviations: AA, arachidonic acid; DHA, docosahexaenoic acid; DHEA, dehydroepiandrosterone; EPA, eicosapentaenoic acid; PUFAs, polyunsaturated fatty acids.

### 4.1 ω‐3 PUFAs

By lay terms, ω‐3 PUFAs are seen as fundamental for a healthy lifestyle, promoting leanness, reducing cardiovascular risk, and increasing longevity.^133^, ^134^ In developmental neurobiology, ω‐3 PUFAs are known to mediate, and even directly control, the balance between self‐renewal (“pro‐survival” effects) and differentiation in embryonic neural stem (NSCs) and progenitor cells.^126^ Particularly, DHA has been reported to affect NSC proliferation, cell–fate decision, and the survival of newly–born progeny by being a major structural constituent of plasma membranes.^126^, ^127^, ^135^, ^136^ During neuronal differentiation, DHA is particularly enriched in growth cones and synaptosomal membranes, whereby the dynamics of DHA availability and exchange with other PUFAs can affect directional growth processes, as well as the excitability of synapses^127^, ^137^ through tuning membrane fluidity and receptor accessibility.^137^ DHA also affects the transcriptional control of the cell‐cycle through *Hes1*, *p27kip1*, *NeuroD*, and *Map2*, which positively affect the expression of terminal targets (e.g., *Camk2*, *Bdnf*, *Syt1*, AMPA/NMDA receptor subunits).^126^, ^136^, ^137^ Consensus view implies that DHA interrupts the cell‐cycle, thus driving NSC‐to‐neuroblast transition.^126^ At the same time, DHA possesses anti‐apoptotic and antioxidant properties, thus increasing the survival of differentiating progeny (alike neuroprotection in adult brain).^129^, ^131^, ^138^

EPA acts on a pool of transcription factors overlapping with those activated by DHA, yet with its effects being markedly different: for example, while DHA significantly decreases *Hes1* expression to promote neuronal differentiation, EPA upregulates *Hes1* instead. Moreover, *Hes6*, which promotes neuronal differentiation by cooperating with *Hes1* in a positive‐feedback loop,^139^ is sensitive to EPA but not DHA. Even though these observations are, to a large extent, limited to in vitro cellular models (Table 5), we note that the levels of EPA and DHA present in embryonic tissues at any time during the expansion of the neuronal pool (that is, from gestational week 5 in humans, and embryonic day 8/9 in mouse), can contribute to the control of complex and fate‐restricting transcription factor matrices to regulate NSCs.^126^, ^136^

It is worth noting that ω‐3‐to‐ω‐6 PUFA conversion, or recruitment, is a prevailing phenomenon affecting NSC turnover and fate choices. We illustrate this by the finding that the effect of EPA on NSC proliferation can be abolished by prior exposure of NSCs to antagonists of either the CB_1_R or CB_2_ cannabinoid receptor (CB_2_R).^90^, ^127^ This is because EPA administration increases tissue levels of AA, which are then partly converted into 2‐arachidonoylglycerol (2‐AG), an efficacious endocannabinoid, whose action at CB_1_R or CB_2_R triggers MAPK signaling. This mechanism is attenuated by both AM251 and AM630, respective CB_1_R and CB_2_R antagonists (see below). Likewise, DHA exposure increases endocannabinoid levels and downstream MAPK activity. Yet, this coupling is ineffective to drive NSC proliferation, and insensitive to either CB_1_R or CB_2_R antagonists.^127^ Here, a crosstalk between DHA and endocannabinoid‐mediated signaling pathways could be suggested by DHA being converted, at least in part, into N‐docosahexaenoylethanolamide (DHEA). DHEA belongs to the *N*‐acetylated amino acid or neurotransmitter class of lipid signaling molecules (designated as “n‐3 endocannabinoid”^19^), including a shared biosynthetic mechanism with arachidonoylethanolamine.^69^, ^140^ DHEA induces neuronal differentiation (neuritogenesis, synaptogenesis)^138^ by activating protein kinase A (PKA)/cAMP response element binding (CREB) signaling through GPR110.^141^ Thus, the observations available to date suggest that the availability of ω‐3 PUFAs is beneficial for neuronal maturation.

### 4.2 ω‐6 PUFAs

“Western” diets are particularly enriched in ω‐6 PUFAs, with their absolute amounts directly linked to obesity.^142^ AA is one of the most abundant ω‐6 PUFAs in the nervous system along the entire lifespan. As the brain evolves, AA concentrations rapidly rise. This is not surprising if one considers the many modes of AA action during cell division,^18^ and intercellular signaling (e.g., synaptic neurotransmission) by itself or through its metabolites that affect protein kinases, ion channels, and SNARE proteins (e.g., syntaxin‐3) that modulate neurite outgrowth, repair, and neurotransmitter exocytosis.^68^, ^143^

AA is a precursor to many bioactive lipids, including endocannabinoids.^68^, ^143^ Endocannabinoids control each of the seven consecutive steps of neuronal development (neurogenesis, survival of progeny, neuroblast migration, neurite outgrowth, axon fasciculation, synaptogenesis, astroglial recruitment and (metabolic) interplay)^144^, ^145^, ^146^, ^147^, ^148^, ^149^ through CB_1_R and/or CB_2_R, and likely GPR55.^143^ Even though multiple endocannabinoids exist (e.g., 2‐AG, anandamide), their redundancy is essential to maintaining an “endocannabinoid tone” to allow neurons (but also glia) to progress through their developmental programs. The fundamental nature of endocannabinoid availability and signaling during brain development is best exemplified by their ligand and/or receptor switches in proliferative vs. differentiating niches in the brain,^144^, ^146^, ^147^, ^150^ the ubiquitous upregulation of *Cnr1*/CB_1_R expression upon neurogenic fate decisions in all prospective neurons,^147^, ^151^ and the partitioning of endocannabinoids to advancing growth cones in a metabolically‐controlled fashion.^144^, ^152^ Besides neurons, AA (as the precursor of adrenic acid, one of the most abundant fatty acids found early in brain development,^153^ and 2‐AG) is also involved in axonal myelination^154^, ^155^, a process whose successful completion determines the speed of action potential propagation along each axon.

**FIGURE 3:**
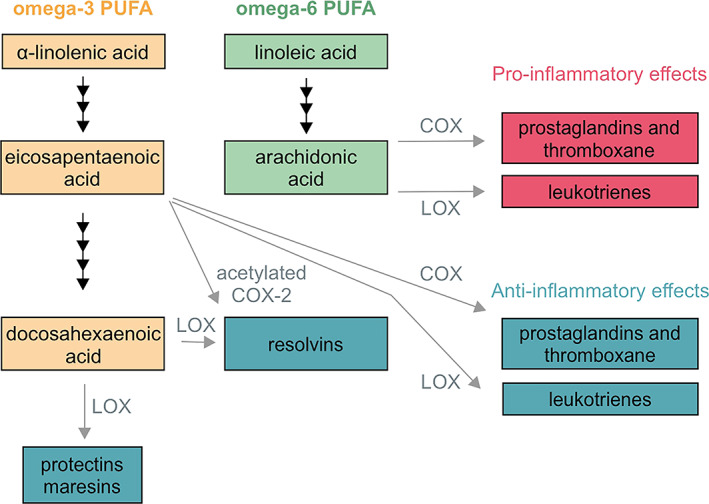
Metabolic pathways converting PUFAs into pro‐, as well as anti‐inflammatory mediators. Cyclooxygenase (COX) and lipoxygenase (LOX)‐dependent enzymatic processes are in grey.

AA/endocannabinoid metabolism (both breakdown and conversion into other eicosanoids) involves cyclooxygenase‐2 (COX2). COX2 is a target for acetaminophen (paracetamol),^156^ as well as non‐steroidal anti‐inflammatory drugs^157^ (Figure 3). The above pathways are therefore likely to be sensitive to the maternal use of “over‐the‐counter” medication to fight, for example, infections, during pregnancy. Thus, care should be exercised when setting dosage regimens for pregnant women because of potential unwanted effects on fetal development, with drug sensitivity (tissue enrichment/accumulation in brain, metabolism, intermediates) likely different from that in the mother. Overall, we suggest that ω‐6 PUFAs are as important as ω‐3 PUFAs to physiological developmental processes, with their actions being complementary to those of ω‐3 PUFAs.

### 4.3 ω‐6:ω‐3 PUFA disbalance in neuronal differentiation

The above mechanisms outline why both ω‐3 and ω‐6 PUFAs are critical for cellular differentiation, and suggest that their gross imbalance in maternal diets during pregnancy could be detrimental for fetal brain development.^88^ We have recently shown that excess ω‐6 PUFA intake during gestation is harmful because of the ensuing deregulation of endocannabinoid signaling, which yields CB_1_R loss‐of‐function by desensitization.^9^ Notably, this mechanism is phenotypically similar to *Cnr1* ^−/−^ mice and to gestational THC exposure,^158^ with axonal wiring defects, and anxiety and depression‐like traits in adult offspring.^9^

The importance of keeping a balanced diet is best explained by the existence of compensatory and convergent metabolic pathways to safeguard PUFA availability (Figure 1). Accordingly, when nutritional uptake of ω‐3 PUFAs is particularly poor, surplus ω‐6 PUFA derivatives are produced by the endoplasmic reticulum and peroxisomes to replace ω‐3 PUFAs in, for example, biological membranes.^18^ Consequently, neurons with reduced somatic sizes appear,^159^ with their polarized dendritic structures also significantly shrunken.^160^ At the molecular level, insufficient ω‐3 PUFAs reduce the expression of vesicular neurotransmitter transporters, thus depleting the content of presynaptic vesicles and limiting synaptic neurotransmission.^25^, ^160^ Moreover, changing the dietary EPA:DHA ratio (noting that DHA is likely unable to cross the placenta and is in situ regenerated^24^) directly impacts EPHA:DHEA levels,^69^, ^138^ whose misalignment (given a significant excess of EPA) could lead to neuronal misplacement and the arrest of neuritogenesis. Overall, we propose that balanced ω‐3: ω‐6 PUFA intake is necessary for correct neuronal development instead of disproportionately increasing nutritional ω‐3 PUFA availability for pregnant women.

## 5 BEYOND NEURONS: PUFAS IN ASTROGLIA SAFEGUARD METABOLIC HOMEOSTASIS

Neurons develop in the proximity of glial cells. Astrogliogenesis is largely subsequent to neurogenesis, peaking at the early neonatal period in mice.^161^ Astrocytes are considered to provide circuit‐specific metabolic support to neurons and are integral to “tripartite synapses” with their leaflets enriched in neurotransmitter transporters and enzymes to maintain the temporal and spatial fidelity of synaptic neurotransmission. Microglia are immune lineage‐derived cells, considered fundamental for immune surveillance, pruning of synapses, and modulating inflammatory responses upon disease.^161^ Both astrocyte metabolism and microglial immunosurveillance are affected by dietary PUFAs. Here, we focus on astrocytes because of the prevalence of mechanistic and developmental biology data (Table 6). For microglia, most studies on their ω‐3/ω‐6 PUFA‐selective transformation, ligands/mediators (e.g., resolvins, protectins, and maresins; Figure 3), receptor‐mediated signaling events, and cytokine cascades are available from adult models (including adult neurogenesis and sex‐specific effects), for which we direct the reader to comprehensive reviews.^160^, ^162^, ^163^, ^164^, ^165^, ^166^, ^167^

**TABLE 6:** Astrocyte functions affected by either ω‐6 PUFAs or ω‐3 PUFAs; in vitro and in vivo experiments.

Experimental procedures	Species	Molecular/cellular outcomes	References
DHA treatment of cortical astrocytes	Rat	↑ gap junction coupling by redistributing functional connexin‐43,	Champeil‐Potokar et al.^169^
DHA treatment of cortical astrocytes	Rat	↓ activity of glutamate transporters (GLAST and GLT1), ↓ glutamate uptake.	Grintal et al.^170^
DHA treatment of cortical astrocytes	Mouse	inhibition of Ca^2+^ depletion of endoplasmic reticulum, attenuated endoplasmic reticulum stress and cell death.	Begum et al.^174^
DHA treatment of cortical astrocytes	Rat	↑ transcription and endocytosis of α_2_‐adrenergic receptors, ↑ ERK signaling for differentiation.	Das et al.^176^
DHA and EPA treatment of patient‐derived stem cells	Human	↑ GFAP expression, ↑ CREB activity.	Yu et al.^177^
AA, DHA and EPA treatment of cortical astrocytes	Rat	reduced thrombin‐induced Ca^2+^ oscillations, decreased intracellular Ca^2+^ content.	Sergeeva et al.^173^
AA and DHA treatment of cortical astrocytes	Rat	DHA (but not AA) prevents corticosterone‐induced glutamate uptake, DHA decreases glutamine synthetase activity, DHA delays corticosterone‐induced cytoskeletal alterations.	Champeil‐Potokar et al.^169^
AA and derivatives in vitro and in vivo	Mouse	↑ progenitor proliferation and differentiation into astroglia.	Aguado et al.^149^
LA treatment of neural progenitors	Mouse	↑ GFAP expression, ↑ GABA‐driven astroglial differentiation.	Shinjyo et al.^180^
Maternal **ω**‐3 deficient diet	Rat	↓ DHA in membrane phospholipids in cortex, hippocampus, hypothalamus, ↓ glucose uptake, ↓ brain glucose transporter GLUT1.	Ximenes da Silva et al.^171^ Pifferi et al.^172^
Hippocampal slices	Mouse	Endocannabinoids activate astroglial cannabinoid receptors, Endocannabinoids increase Ca^2+^ levels and glutamate release.	Navarrete et al.^178^, ^179^

Abbreviations: AA, arachidonic acid; DHA, docosahexaenoic acid; EPA, eicosapentaenoic acid.; PUFAs, polyunsaturated fatty acids.

Astrocytes are the only cell type in the brain with a capacity to synthesize DHA^168^ which, once produced, can be used to supply neurons.^168^ The role of DHA in astrocytes during brain development is incompletely understood, with recent reports claiming that DHA facilitates astrocyte coupling by gap junctions,^169^ glutamate^170^ and glucose uptake,^171^, ^172^ the regulation of Ca^2+^ signaling,^173^ and the inhibition of endoplasmic reticulum stress.^174^ Moreover, cell biology studies point out the ability of DHA to help astrocytes to resist stress,^175^ and to promote their differentiation.^176^ Astrocyte differentiation is also facilitated by EPA.^177^ Our data suggest that slowed protein turnover through the unfolded protein response (UPR) pathway is central for EPA (and/or EPEA)‐mediated effects, and guides astroglia‐to‐neuron signaling.

Cannabinoid receptors in astroglia^178^, ^179^ provide partial, yet important, signaling routes for AA metabolites. Most notably, endocannabinoids define cell fate decisions, that is, if NSCs generate neurons or astrocytes. Herein, the activation of CB_1_Rs promotes NSC fate choice towards astrocytes.^149^, ^180^ Moreover, astrocytic CB_1_Rs maintain lactate flow from astroglia to neurons, thus directly impinging on synaptic signaling.^178^, ^179^ In NSCs in vitro, LA and practically all downstream ω‐6 PUFAs upregulate genes relevant for endocannabinoid signaling, which results in a sustained enhancement of 2‐AG bioavailability. Considering that 2‐AG stimulates astrogliogenesis in a CB_1_R‐dependent manner,^180^ it is plausible that ω‐6 PUFA‐enriched maternal diets during gestation and lactation can exert notable impact on the number of astrocytes and their recruitment to functionally‐devolved neuronal micro‐, meso‐, and macrocircuits.

## 6 THE EFFECT OF PUFAS ON CELLULAR BIOENERGETICS

Proliferation and differentiation are energy‐consuming processes for NSCs during both embryonic and adult neurogenesis. Likewise, the migration, differentiation, synapse maintenance, and signaling of postmitotic neurons are amongst the peak energy‐demanding cellular features of the body.

The main cellular organelles providing bioenergy are the mitochondria, which use the mitochondrial oxidative phosphorylation (Oxphos) cascade to produce ATP.^181^ There is a substantial body of clinical epidemiological data demonstrating that neurodevelopmental disorders are accompanied by mitochondrial dysfunction.^181^, ^182^ Maintaining mitochondrial function is also important for NSC commitment and fate decision.^183^ A reduction of NSC self‐renewal and proliferation, defects in cell cycle exit, and ensuing neuronal differentiation have been found following mitochondrial dysfunction.^184^

A variety of cellular stressors can compromise mitochondrial function and integrity, with PUFA‐imbalanced diet during pregnancy considered a significant risk factor.^185^ ω‐3 PUFA intake could improve mitochondrial functions, including their fusion, and reduces the production of reactive oxygen species (ROS). In particular, DHA and EPA promote mitochondrial biogenesis and improve the expression of genes associated with ATP production and energy metabolism.^185^, ^186^ Moreover, DHA from dietary sources is rapidly incorporated into mitochondrial membranes, where it participates in the Oxphos cascade to produce ATP.^187^ Thus, ω‐3 PUFA deficiency could alter the course of brain development not only through the perturbation of the biophysical integrity of cellular membranes but also by suppressing mitochondrial energy yield.

In contrast, ω‐6 PUFAs attenuate mitochondrial respiration and reduce the mitochondrial membrane potential.^188^ Again, endocannabinoids are amongst the ω‐6 PUFA derivatives that modulate mitochondria. In neurons, CB_1_Rs partitioned to mitochondria reduce mitochondrial respiration by inhibiting PKA signaling, and Oxphos activity (at the level of complex I), resulting in lower cellular ATP reserves.^189^ Most recently, CB_1_Rs were also localized to astrocytes,^190^ in which endocannabinoid mobilization promoted mitochondrial Ca^2+^ signaling, and facilitated astrocyte‐to‐neuron lactate transport^191^ to maintain synaptic activity and plasticity. Thus, ω‐3/ω‐6 PUFAs could affect mitochondrial integrity and functions in both neurons and astrocytes, and shape intercellular communication by metabolic coupling.

## 7 FUTURE PERSPECTIVES

Considering the ever‐increasing prevalence of being overweight or obese during pregnancy, and the mechanistic data that had accumulated during the past decades, we await a series of molecular breakthroughs to dissect how maternal nutrition shapes the many cellular contingents of the fetal brain. From a clinical perspective, we propose a focus on the ω‐6: ω‐3 PUFA balance of maternal diets, rather than the dominance of either ω‐3 or ω‐6 PUFAs. Novel insights will not only justify the rationale of weight control during pregnancy but also aid the identification of when, where, and how medical interventions can mitigate the adverse effects of gestational dietary PUFA imbalance on the offspring's brain.

Among the emerging fields of intergenerational metabolomics, we see great potential in integrating diet‐induced alterations in the composition of the maternal gut microbiome with the known effects of microbiome‐derived bioactive mediators of neuronal and glial differentiation. Given that the microbiome dynamically drives gene–environment interactions, and exerts a substantial influence on brain function and behavior during all stages of life,^192^ understanding how specific PUFAs (and/or their bioactive derivatives) impact the acquisition of cellular identity in the fetal brain appears to be a fascinating endeavor.

Regardless of the molecular mediators, persistent epigenetic alterations seem appealing to code life‐long and generation‐spanning consequences of maternal dietary choices. Any such mechanism might involve the early reprogramming of primordial germ cells in the developing fetus, not only somatic lineages, thus influencing subsequent generations. Pharmacological developments and trials with, for example, histone deacetylase inhibitors, might be of medical benefit. Alternatively, non‐epigenetic routes possibly affecting offspring neurodevelopment may include differences in the macro‐ and micronutritional value and composition of the mother's milk,^193^ alterations in maternal care, or other/additional pathways of behavioral transfer of traits of social, emotional, and cognitive functions.^194^ Therefore, novel insights in PUFA action on neurodevelopment could have a beneficial impact on the quality of life of affected children.

## AUTHOR CONTRIBUTIONS

**Valentina Cinquina:** Conceptualization; data curation; formal analysis; investigation; writing – original draft. **Erik Keimpema:** Conceptualization; funding acquisition; writing – review and editing. **Daniela D Pollak:** Conceptualization; funding acquisition; writing – review and editing. **Tibor Harkany:** Conceptualization; funding acquisition; writing – review and editing.

## FUNDING INFORMATION

This work was supported by the Austrian Science Fund (FWF, P 34121‐B to E.K.; I 4854 and P 34281 to D.D.P.), the Swedish Research Council (2018‐02838; to T.H.), the Swedish Brain Foundation (Hjärnfonden, FO2020‐0178, to T.H.), the Novo Nordisk Foundation (NNF20OC0053667, to T.H.), the European Research Council (FOODFORLIFE, ERC‐2020‐AdG‐101021016; to T.H.), and intramural funding from the Medical Neuroscience Cluster of the Medical University of Vienna (2021‐1 to T.H.).

## CONFLICT OF INTEREST STATEMENT

The authors of this manuscript declare no conflict of interest.

### PEER REVIEW

The peer review history for this article is available at https://www.webofscience.com/api/gateway/wos/peer-review/10.1111/jne.13320.

## Data Availability

Data sharing is not applicable to this article as no new data were created or analyzed in this study.
